# From the Popoveniuc Score to Therapeutic Protocols: A Comprehensive Review of Myxedema Coma

**DOI:** 10.7759/cureus.101023

**Published:** 2026-01-07

**Authors:** Rodolfo A Valerio Aguirre, Karla C Ocaña Martinez, Erick U Martinez Rodriguez, Taide I Cabrera López, Jonathan G Gonzalez Mena

**Affiliations:** 1 Emergency Medicine, Hospital Beneficencia Española De San Luis Potosí, San Luis Potosi, MEX; 2 Internal Medicine, Hospital Regional De Alta Especialidad Dr. Ignacio Morones Prieto, San Luis Potosi, MEX; 3 General Medicine, Universidad Autónoma De San Luis Potosí, San Luis Potosi, MEX; 4 General Medicine, Hospital Beneficencia Española De San Luis Potosí, San Luis Potosi, MEX

**Keywords:** combination therapy, diagnostic score, myxedema coma, replacement therapy, severe hypothyroidism

## Abstract

Myxedema coma represents the most severe and life-threatening manifestation of hypothyroidism, characterized by multiorgan dysfunction, which leads to high mortality. Its incidence is low, estimated at less than one case per million inhabitants, but it constitutes an endocrinological emergency of great clinical relevance. The pathophysiology is related to the loss of compensatory mechanisms in response to precipitating factors, triggering neurological, cardiovascular, respiratory, and metabolic alterations. Diagnosis is often delayed due to the nonspecific nature of initial symptoms and the fact that serum thyroid hormone concentrations do not accurately reflect the severity of the condition. In this context, clinical tools such as the Popoveniuc scale have become valuable in supporting diagnostic suspicion. Early recognition and timely initiation of treatment with hormone replacement therapy and intensive supportive care are essential to improve prognosis and reduce complications, underscoring the importance of increased clinical awareness in emergency and critical care settings.

## Introduction and background

Myxedema coma is an endocrinological emergency, characterized by a high mortality rate of between 30% and 50% [1]. The annual incidence of myxedema coma in Western countries is estimated to be approximately 0.22 cases per million people [2,3]. This pathology is more common in women and in individuals over 60 years of age, particularly during winter months [4]. However, recent studies have reported a shift toward an earlier age of onset, with newer cases presenting around 40 years of age [5].

The term myxedema coma was coined by the English physician Vincent Summers in 1953, after documenting four fatal cases in patients presenting with clinical features of myxedema in a comatose state [2]. The thyroid gland is an endocrine organ located in the anterior part of the neck, at the level of the second and third tracheal rings. It weighs between 10 and 20 grams in adults and is highly vascularized, receiving blood supply from the superior and inferior thyroid arteries, with venous drainage through the superior, middle, and inferior thyroid veins [3]. Its anatomical location and endocrine function contribute to its critical physiological importance.

This article is a narrative review of the literature on myxedema coma. Searches were conducted in PubMed, Scopus, and Google Scholar to identify relevant articles published in recent years, using combinations of the terms “myxedema coma,” “severe hypothyroidism,” “diagnostic scores,” “Popoveniuc score,” “thyroid hormone replacement,” “levothyroxine,” and “liothyronine.” Clinical trials, observational studies, case series, case reports, and review articles addressing the pathophysiology, clinical manifestations, diagnostic tools, and treatment strategies for myxedema coma in adult patients were included. The review focuses on highlighting clinically relevant findings and areas of uncertainty to support the proposed diagnostic and therapeutic algorithm for patients presenting to the emergency department.

## Review

Physiology

Thyrotropin-releasing hormone, produced by the hypothalamus, acts on the anterior pituitary gland to induce the release of thyroid-stimulating hormone (TSH). This hormone, in turn, triggers a series of intracellular signaling pathways, including cyclic adenosine monophosphate (cAMP), phospholipase C, and protein kinase A, which are involved in various processes that regulate the function and activity of thyroid hormones [6].

The production of cAMP and protein kinase A aids in iodide uptake and the activity of the sodium iodide symporter, whereas phospholipase C signaling primarily regulates iodide efflux and hydrogen peroxide production. The sodium iodide transporter moves two sodium ions along with one iodide molecule into the thyroid follicular cell, while the sodium concentration gradient is formed by sodium-potassium ATPase, which actively pumps sodium ions out of the follicular cell cytoplasm. Iodide within the follicular cell is oxidized by thyroid peroxidase and used in the iodination of thyroglobulin, which is subsequently degraded to release tetraiodothyronine (T4) and triiodothyronine (T3) [6].

Thyroid hormones, especially T4, are transformed into T3 in peripheral tissues through the loss of an iodine molecule by the enzyme iodothyronine deiodinases: type I in the liver, kidney, and thyroid; type II in the brain, pituitary gland, and thyroid; and type III in the placenta and fetal tissues. Although T3 is more potent, it has a shorter half-life than T4 due to its lower affinity for plasma proteins. These hormones exert their effects by entering almost all cells of the body, where T3 binds to nuclear receptors that regulate genetic transcription. This interaction activates or inhibits the expression of specific genes, resulting in increased synthesis of proteins essential for enzymatic, structural, and cellular transport functions. The overall effect of thyroid hormones is increased functional activity, which is observed in various organ systems [7].

Pathophysiology

Myxedema coma is the most severe manifestation of untreated hypothyroidism. It involves severe and prolonged depletion of thyroid hormones, resulting from various diseases or non-thyroidal factors that cause widespread systemic involvement. The term "myxedema coma" may be misleading, as most patients do not present with coma at the onset of the disease [8,9].

This condition relies on adaptive neurovascular mechanisms, which are lost in response to a triggering factor that causes an abnormal decrease in T4 and, subsequently, a decrease in peripheral intracellular T3, with a resulting elevation of TSH (in the case of primary hypothyroidism) or a normal or low concentration of TSH (in the case of secondary hypothyroidism). Laboratory-measured changes in thyroid hormone levels do not predict the severity of the condition, as patients with elevated TSH can compensate for a hypothyroid state, whereas other patients with minimal TSH elevations can still develop the disease [10,11].

Precipitating Factors

Infections and sepsis, including pneumonia, urinary tract infections, and bacteremia, are among the main precipitating factors for myxedema coma. Other triggers include stroke, congestive heart failure, gastrointestinal bleeding, exposure to cold, and the use of various medications such as analgesics, antidepressants, antipsychotics, amiodarone, and sedatives [12]. While the pathophysiology of this condition is primarily attributed to low levels of intracellular T3, the literature does not identify discontinuation of thyroid hormone replacement therapy as a precipitating factor in this population [13].

There are significant repercussions on the central nervous system, where thermogenesis is dysregulated, leading to hypothermia (<35° C). If this temperature persists, an altered mental status ensues. In the respiratory system, ventilatory insufficiency occurs with increased CO₂ (carbon dioxide) concentrations and hypoxemia, resulting in cerebral anoxia, which may require mechanical ventilation and intubation [14-17].

Cardiovascular involvement includes arrhythmias and bradycardia (<60 beats/minute), which occur due to decreased inotropism, with a reduction in stroke volume and hypotension (<90/60 mmHg), leading to cardiogenic shock [18].

Finally, decreased vascular permeability is observed, resulting in reduced renal excretion of free water and increased secretion of antidiuretic hormone, causing edema and hyponatremia [14,19].

Clinical manifestations

**Figure 1:**
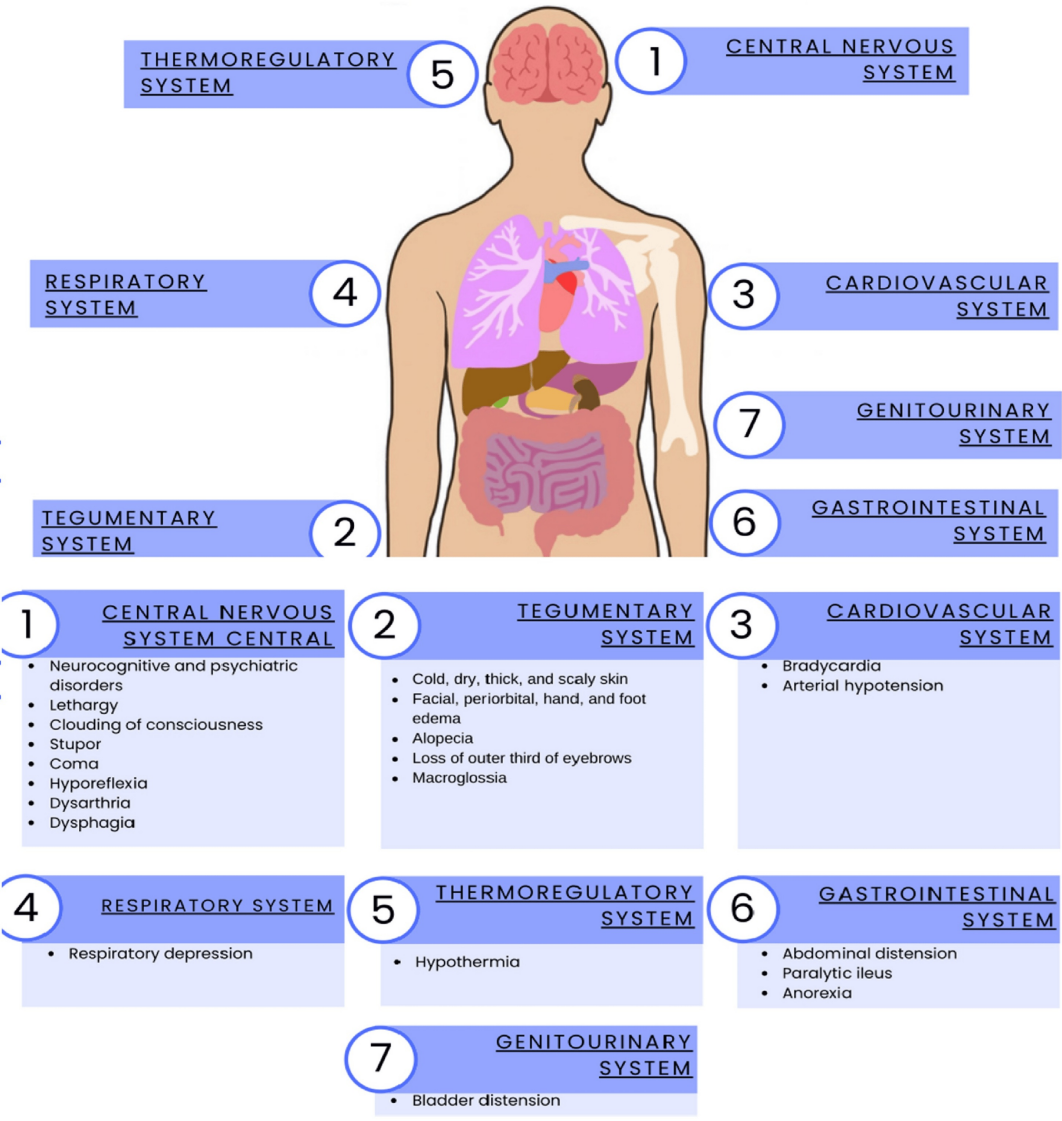
Clinical manifestations of myxedema coma by organ system Adapted from Pérez-Nieto OR, et al. [17]. Schematic representation of the main clinical manifestations of myxedema coma, grouped by organ system: (1) central nervous system: neurocognitive and psychiatric disorders, lethargy, clouding of consciousness, stupor or coma, hyporeflexia, dysarthria, dysphagia; (2) tegumentary system: cold, dry, thick, scaly skin; facial, periorbital, hand, and foot edema; alopecia; loss of the outer third of the eyebrows; macroglossia; (3) cardiovascular system: bradycardia and arterial hypotension; (4) respiratory system: respiratory depression; (5) thermoregulatory system: hypothermia; (6) gastrointestinal system: abdominal distension, paralytic ileus, anorexia; (7) genitourinary system: bladder distension. Image credit: Created in Microsoft Word (Microsoft® Corp., Redmond, WA) by the authors.

Thyroid hormones act on multiple organs and metabolic functions of the body. The most common manifestations of myxedema include neurological impairment and hypothermia, but it can often be accompanied by bradycardia, hypoventilation, and hypotension [20-22]. However, the clinical presentation is usually nonspecific and requires a high degree of clinical suspicion. Progression is typically gradual, occurring over weeks or months (Figure 1) [20].

Neurological Manifestations

Contrary to what the term may suggest, coma is not typically present; rather, patients exhibit an altered state of consciousness ranging from lethargy and confusion to stupor, apart from memory loss, ataxia, psychosis (myxedema psychosis), and seizures, which may also be present. Sensory and motor peripheral neuropathy, ataxia, and uncoordinated movements of the hands and feet may also occur. Metabolic alterations are also observed, such as elevated levels of antidiuretic hormone accompanied by decreased urine output. Physical examination reveals diminished deep tendon reflexes during relaxation. All of this is due to decreased cerebral blood flow and impaired glucose metabolism in the brain [4,23,24].

Cardiovascular Manifestations

Thyroid hormones play an important role in regulating blood pressure. Patients with hypothyroidism tend to have reduced cardiac output and systolic hypertension [25]. However, in cases of severe hypothyroidism or myxedema coma, decreased cardiac output, bradycardia, and diastolic hypotension occur due to reduced oxygen demand in cardiac tissue [25].

These patients may also present with cardiomegaly, which may be secondary to ventricular dilation or pericardial effusion, leading to heart failure [26]. Recent evidence highlights the relevance of cardiovascular dysfunction in myxedema coma. Vázquez-Fuster et al. reported a case of severe hypothyroidism with bradycardia and left ventricular systolic dysfunction that resolved after hormonal and hemodynamic support, demonstrating the reversibility of cardiac involvement [27].

This condition is accompanied by electrocardiographic abnormalities such as low voltage, QT interval prolongation, and ST-segment changes [4].

Respiratory Manifestations

Centrally, these patients present with respiratory depression and little to no response to hypoxemia and hypercapnia, exhibiting respiratory acidosis, which may require mechanical ventilation [28]. Furthermore, the presence of goiter, macroglossia, pharyngeal edema, and muscle weakness may contribute to airway obstruction [28,29].

Dermatological Manifestations

Dermatological changes reflect the profound systemic slowing of metabolism. The accumulation of mucopolysaccharides, hyaluronic acid, and chondroitin sulfate in the skin and subcutaneous tissue contributes to the myxedematous appearance. Due to their water-absorbing properties, these substances produce non-pitting edema, resulting in periorbital swelling, lip thickening, acral swelling, and tongue enlargement [30]. In addition, patients often present with dry, rough, and brittle scalp and body hair, which may progress to partial or complete alopecia [31]. Hair loss from the lateral third of the eyebrows is a characteristic finding. The rate of hair growth is markedly reduced, probably due to reflex vasoconstriction secondary to decreased metabolic activity and reduced skin perfusion. The skin texture is usually rough, cold, and pale, with xerosis and scaling [30,31].

Renal Manifestations

A decrease in glomerular filtration rate and renal plasma flow may occur, and hyponatremia is common, secondary to an increase in antidiuretic hormone [32].

Depending on the degree and severity of hyponatremia, it may contribute to altered mental status, and when severe (105-120 mEq/L), it can be largely responsible for precipitating a comatose state in this condition [33].

Adrenal Manifestations

This condition may occur due to autoimmunity secondary to Hashimoto's thyroiditis with adrenal insufficiency, or secondary to hypothalamic-pituitary insufficiency, in which decreased gluconeogenesis due to hypocortisolism results in hypoglycemia, hyponatremia, and hypotension [34].

Hematologic Manifestations

Severe hypothyroidism produces a hypercoagulable state and an increased risk of bleeding associated with acquired von Willebrand syndrome type 1 (decreased levels of factors V, VII, VIII, IX, and X), which is reversible upon initiation of levothyroxine replacement therapy [35].

Other causes of bleeding in these patients are associated with sepsis, which can lead to disseminated intravascular coagulation. Microcytic anemia secondary to bleeding or iron deficiency anemia may also contribute to altered neurological status [29,35].

Gastrointestinal Manifestations

These patients present with neuropathic changes that cause gastric atony, impaired peristalsis, and paralytic ileus. Among other complications, ascites and gastrointestinal hemorrhage may occur due to the associated coagulopathy [36,37].

Myxedema coma should be suspected in patients with a comatose state or sensory disturbances such as hypothermia or hypercapnia, with or without a history of thyroid dysfunction. Therefore, it is suggested that all patients presenting with neurological deficits and these characteristics should initially undergo a thyroid profile [38,39].

Furthermore, a thorough medical history is crucial, including information on thyroid dysfunction, goiter, thyroidectomy, cervical radiotherapy, pituitary surgery and radiotherapy, head trauma, and postpartum bleeding [38,39].

Laboratory studies

Since more than 95% of myxedema coma cases are due to primary hypothyroidism, laboratory findings include elevated serum TSH levels and low or undetectable serum concentrations of total and free T4 [17,40]. Administration of certain drugs such as dopamine or glucocorticoids also decreases TSH levels. T4 and T3 concentrations (total and free fractions) will always be low [32,40].

Measurement of cortisol and ACTH is also important, as secondary adrenal insufficiency may be associated in 5-10% of cases [40].

It is worth noting that this disease affects multiple organs; therefore, patients may present with other associated laboratory abnormalities, such as hyponatremia and hypoglycemia. Anemia, leukopenia, and elevated LDH and CPK levels associated with myopathy may also be present. Arterial blood gas analysis may reveal hypoxemia, hypercapnia, and acidosis [21,32].

Evaluation/diagnosis systems

The diagnosis of myxedema coma in the emergency department is often difficult, as patients usually present with altered mental status and an insufficient medical history. Furthermore, its manifestations can be similar to those of more prevalent conditions, such as sepsis, metabolic encephalopathy, or cerebrovascular events [20,31].

**Figure 2:**
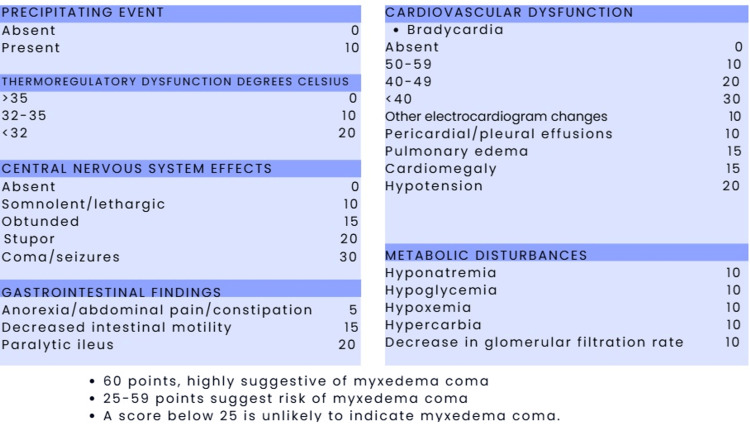
Popoveniuc diagnostic scoring system for myxedema coma Adapted from Popoveniuc G, et al. [31]. Scoring scheme for myxedema coma based on six domains: precipitating event, thermoregulatory dysfunction (°C), central nervous system effects, gastrointestinal findings, cardiovascular dysfunction, and metabolic disturbances. Each clinical or laboratory abnormality is assigned a specific point value; the total score is obtained by summing all applicable items. A total score of 60 points is considered highly suggestive of myxedema coma, 29-59 points indicate a risk of myxedema coma, and 25 points make the diagnosis unlikely.

There are different tools for establishing the diagnosis, including a more specific scale for myxedema coma created by Popoveniuc (Figure 2). This scale assesses and scores the degree of hypothermia, cardiovascular, gastrointestinal, metabolic, and neurological involvement, and the presence or absence of a triggering event or factor [38].

Furthermore, diagnostic scoring systems such as the BWPS and JTA criteria, extensively analyzed by Kruithoff and Gigliotti (2025), remain essential for differentiating severe hypothyroidism from myxedema coma [9]. Although the Popoveniuc diagnostic scoring system for myxedema coma has a sensitivity of 100% and a specificity of 85% [31], biases exist regarding the clinical criteria this scale evaluates, since in cases of myxedema coma reported in the literature, some characteristics were mild or absent. Altered mental status is a prominent aspect of the clinical presentation in case reports; however, it would be difficult to base the diagnosis solely on this finding, as numerous pathologies can cause altered neurological status. We recommend that in the emergency department, patients for whom information about previous illnesses is unavailable or who do not have a prior diagnosis of hypothyroidism, other signs and symptoms should always be considered to rule out more prevalent diagnoses.

Treatment

When myxedema coma is suspected, treatment should be initiated promptly without waiting for laboratory confirmation in order to replace the deficit that led to the myxedema coma and saturate the circulating thyroid hormone stores [4,31]. Despite early recognition and appropriate treatment, mortality remains high [41,42]. The presence of hypotension, respiratory failure, hyponatremia, and delayed medical attention are independent predictors of a poor prognosis in myxedema coma [33]. The therapeutic approach is based on two key components: the immediate initiation of high-dose levothyroxine replacement therapy and specific treatment of the underlying triggering factor [4,31].

Initial treatment consists of high-dose levothyroxine (T4), 200-400 mcg IV over the first 48 hours as the initial dose. In severe cases, higher doses of 300-500 mcg may also be initiated (these doses should be avoided in elderly patients, those with malnutrition, or those with a history of arrhythmias or myocardial infarction), followed by a dose of 50-100 mcg/day depending on clinical evolution [23,21,42].

In settings where parenteral forms of levothyroxine are unavailable, enteral administration (oral, nasogastric, or rectal) should be considered depending on the patient's clinical condition. There is no significant difference in mortality between intravenous and oral levothyroxine [5,33]. Oral levothyroxine dose=intravenous levothyroxine dose/0.75 [42,43].

Glucocorticoids should also be used before or during levothyroxine administration, as severe hypothyroidism or myxedema coma decreases the adrenal response, and thyroid hormones increase cortisol metabolism, which can lead to adrenal crisis. It is recommended to administer 50 to 100 mg of hydrocortisone intravenously every six hours for seven to 10 days [42,44].

Adverse effects of high doses of levothyroxine include precipitation of angina, heart failure, and arrhythmias. Therefore, treatment should be administered with caution in patients with cardiovascular disease who require continuous cardiac monitoring [23,45].

Treatment is considered successful if the patient shows improvement in neurological status, cardiac and pulmonary function, as well as other clinical and laboratory parameters, observed during the first week. Definitive changes in serum TSH and T4-T3 levels are observed after six to eight weeks. Peripheral thyroid hormone measurements are recommended every one or two days to monitor the response to the established treatment [42].

Tetraiodothyronine/Triiodothyronine Combination Therapy

Some authors propose the use of combination therapy because this condition presents with decreased conversion of T4 to T3 (sick euthyroidism syndrome), making the use of LT4 monotherapy a disadvantage [21,45]. Among the advantages of using T3 are its faster onset of action and the absence of peripheral conversion. However, its disadvantages include a shorter half-life compared to T4 and fluctuating serum levels [45,46].

**Figure 3:**
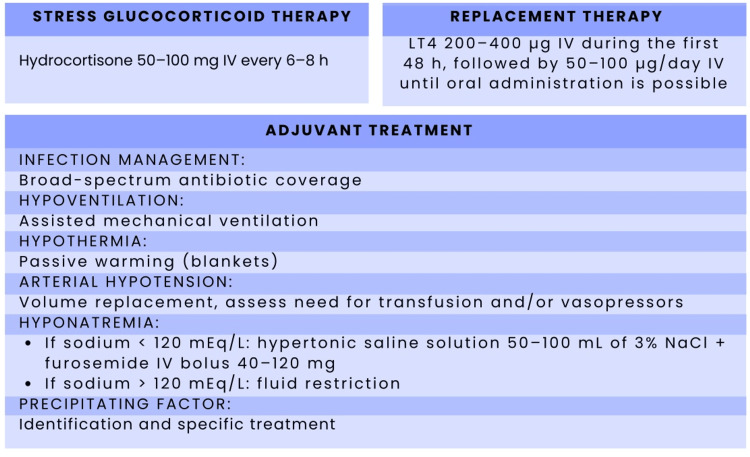
Therapeutic approach for myxedema coma Adapted from Garcés YK, et al. [21]. Summary of the main components of therapy for myxedema coma, including stress glucocorticoid therapy (hydrocortisone 50-100 mg IV every 6-8 hours), thyroid hormone replacement therapy (intravenous levothyroxine 200-400 µg during the first 48 hours, followed by 50-100 µg/day IV until oral administration is possible), and adjuvant treatment. Adjuvant measures comprise infection management with broad-spectrum antibiotic coverage, assisted mechanical ventilation for hypoventilation, passive warming for hypothermia, volume resuscitation and vasopressors for arterial hypotension, stepwise correction of hyponatremia, and identification and specific management of precipitating factors. Image credit: Created in Microsoft Word (Microsoft® Corp., Redmond, WA) by the authors.

Therefore, the concurrent use of LT4+T3 (liothyronine) could be considered, as recommended by the American Thyroid Association (ATA), with an initial bolus dose of 5-20 mcg followed by 2.5-10 mcg every eight hours, and using lower doses in elderly patients and those with cardiovascular disease [46-48]. Liothyronine treatment should be continued until clinical improvement occurs and the patient is stabilized. Excessive liothyronine replacement should be avoided (Figure 3) [46,47].

On the other hand, adding T3 is recommended only if the patient does not respond to LT4 within 24-48 hours of starting treatment [48-50]. Their combined use remains controversial.

## Conclusions

Myxedema coma remains a rare but fatal endocrine emergency. Its prognosis improves with early recognition, the use of the Popoveniuc scale as a diagnostic aid, and immediate initiation of levothyroxine replacement therapy, along with hydrocortisone and individualized intensive care. Close monitoring of temperature, hemodynamics, ventilation, electrolytes, and neurological status allows for dose adjustment and anticipation of cardiovascular complications. Identifying and correcting precipitating factors, comorbidities, and medications contributes to reducing mortality. Persistent uncertainties regarding the optimal use of T3 highlight the need for standardized protocols, prospective research, and training to establish a safe, effective, and timely care pathway.
